# Remarks on the Origin of the Gibraltar Fever of 1828

**Published:** 1831-04

**Authors:** M. Guyon

**Affiliations:** One of the Chief Physicians Attached to the French Army which Occupied Cadiz, in the Years 1827 and 1828.


### GIBRALTAR FEVER.

*Remarks on the Origin of the Gibraltar Fever of* 1828. By M. Guyon, one of the chief Physicians attached to the French Army which occupied Cadiz, in the Years 1827 and 1828.*

Was the Gibraltar disease of 1828 local, or foreign? in other words, was it spontaneously developed, or was it imported? Such are the questions which were raised on the first appearance of that malady; such also are the questions which have been repeated at each new irruption of that scourge into the peninsula.

I shall not enter into any inquiry as to the nature of the disease: it was evidently the yellow fever. No difference of opinion has ever existed on this point; I shall pass it over.

Whether the yellow fever be contagious or not, there is nothing repugnant in the admission that it may develope itself spontaneously; for, as others have said before me, the first time that a contagious disease appeared, it could not have originated in any other manner. Now, granting that the yellow fever may develope itself spontaneously, did the Gibraltar disease originate in this way?

Though the yellow fever were capable of developing itself spontaneously, it would not follow, that it could so develope itself in all places. Many physicians think that it cannot originate spontaneously anywhere, except in America. But, on whatever spots of the globe the yellow fever may be thus developed, still are we completely ignorant as to what the circumstances ought to be, under the influence of which the phenomenon would necessarily be produced. We are well aware that the disease cannot exist anywhere, without a certain degree of heat; but what part would temperature perform in the phenomenon of its spontaneous developement? We know not. The partisans of the spontaneous developement of yellow fever will have temperature play a very active part in its production; some go so far as to assign it no other cause; and on this subject I shall state a fact connected, to a certain degree, with the Gibraltar fever.

During the years 1827 and 1828, I was attached to the Cadiz division. When the disease appeared in Gibraltar, Cadiz and the towns around its bay, (San Fernando, Chiclana, Puerto-Real, Santa-Maria, Rota,) where our division was cantoned, were exposed to the same temperature as Gibraltar. The maximum of that temperature was from twenty-three to twenty-four degrees of the centigrade thermometer. It was under these circumstances, that we were

* Journal Complémentaire, September 1830.

recalled to France. We had to traverse the whole of Andalusia. The first column of our division marched on the 23d September; the second, to which I was attached, near the person of the commander in chief, on the 24th; the third, on the 26th; the fourth, on the 27th. The disease was then raging at Gibraltar, in all its force. Our division, consisting of 7,595 men, was mostly composed of recruits recently arrived from France, and consequently very susceptible to the influence of a new climate. From the 24th of September to the 12th of October, the day on which we crossed the Sierra Morena, we were exposed to a solar heat so scorching, that soldiers fell from the ranks, every day, deprived of sense and motion. The temperature in the sun, to which we were exposed the whole day, (the route we followed not being shaded by a single tree,) was not under from thirty-six to forty centigrade degrees: but still no serious disease appeared amongst us, and some men, whom we left in the hospitals at Carmona and at Cordova, reached France shortly after the division.

The possibility of the spontaneous developement of yellow fever in France, is founded solely on the notion of its identity with the bilious remittent. This is an opinion in which I long participated, and which I renounced only since my residence in Spain. The present being a convenient opportunity, I shall say a few words as to what induced me to adopt a different opinion.

On the 7th of July, 1828, I set out from Cadiz to visit the strait of Gibraltar, having long felt a desire to become acquainted with its localities; I also wished to obtain some information as to the last plague of Tangiers, that of 1828; as also with regard to the epidemic typhus, which had broken out amongst the prisoners at Ceuta and Tariffa, in the early months of the year. Scarcely had I returned from my voyage, when my Gibraltar friends, amongst others Dr. Hennen, chief physician of the garrison, who afterwards fell a victim to the disease, announced to me the commencement of the epidemic. My conclusion from this intelligence was, that the yellow fever and bilious remittent being identical, in my opinion, the former disease ought to shew itself, ere long, in other parts also of Andalusia. From that moment, my whole attention was directed to the bilious remittent, which the division was in the habit of seeing every year, and which, this year, according to my notions, ought to present itself in another form; in other words, ought to afford us, if not an epidemic of yellow fever, at least some cases of that disease: but such was not the fact. Amongst the most severe cases which the remittent exhibited, some resembled yellow fever so strongly, that I almost believed it really did exist, but no case of it occurred. The year before, at the same season, our most serious cases of remittent had produced similar results; but then we had not a disease close by, as in Gibraltar, which could induce us to conclude, that the same atmospheric circumstances were present there, as with us. Not that, with all my belief as to the yellow fever and the bilious remittent being identical, I had failed to perceive well-marked differences between the one and the other; but it must be owned, such is the influence of an opinion already formed, that, of the facts connected with it, we see only the side favorable to that opinion. The differences between the two diseases are many. I shall take the liberty of pointing out some, and shall select them from those symptoms which would seem most calculated to bring the two diseases into the closest resemblance; viz. the vomiting, and the colour of the skin.

1st. In the yellow fever, as well as in the remittent, the vomiting may consist, at first, in the rejection of the food and drink only, then in the throwing up of mucous or bilious matter. But, in the former disease, the matter vomited most generally assumes a black tint, which in the latter it never acquires, and, by the way, the black vomit consists of blood altered by its having remained in the stomach.*

* The matter constituting what is vulgarly called *black vomit*, is found in the stomach or bowels of almost all who die of yellow fever. (Typhus icterodes.) By some this fluid is still supposed to be a peculiar secretion, by others to be the atrabilis, so much talked of, but never accurately described, by the ancient physicians. Foesius, in his translation of the Aphorisms of Hippocrates, has the following remarkable words on this subject: "Quibus ex morbis acutis, aut diuturnis, aut vulneribus, aut alio quocunque modo attenuatis, *bilis atra*, aut velut sanguis niger subierit, illi postridie moriuntur." This is most certainly true as applied to yellow fever. The simple, but beautiful experiment, which Dr. Stephens (in a most interesting paper, read at the Royal College of Physicians last year,) states that he has frequently made, seems fully to bear out the view entertained by M. Guyon, of the nature of black vomit, namely, that it is a hemorrhage. Dr. Stephens often took blood from the heart, and black vomit from the stomach of the same subject, in the West Indies, and, placing the two fluids in separate glasses, could scarcely distinguish one from the other. A solution of Rochelle salts added to each, always changed the colour of both from black to red; the only difference being, that the red assumed by the cardiac blood was brighter than that of the stomachic blood, or black vomit. We hope Dr. S. will follow up his researches so auspiciously begun, and that he will enable us to draw a satisfactory line of distinction between the pseudo black vomit or dark-coloured bilious matter thrown up in some ordinary fevers, and the fatal hemorrhagic discharges from the stomach, in true Typhus icterodes. M. Andral, in his recent work on Pathological Anatomy, considers the black vomit in yellow fever as a pure instance of intestinal hemorrhage, not depending on a primary alteration of the digestive, mucous tissue, but on an altered state of the blood itself, giving it an universal tendency to escape from its vessels.—Translator. (Vide p. 256 of our last number.)

2d. In the yellow fever, the colouring of the skin is owing to the presence of blood which stagnates in the capillaries, or which escapes from them, and is nothing else than the tinge of a contusion, or of any similar lesion; whilst, in the remittent, the colour is owing to the presence of bile, and therefore constitutes a true icterus.

We have seen that the possibility of a spontaneous developement of yellow fever in Europe, is still a problem. But, admitting that it can be so developed in Europe, if the Gibraltar *epidemic* broke out in this way, why did not the disease, at the same time that it appeared there, appear also in all the other towns in the neighbourhood of Gibraltar, such as Estapona and Marbilla to the east, Algeciras and Tariffa to the west, which, besides their proximity to Gibraltar, are placed, as that city is, on the sea coast, and subject to the same atmospheric influences.* Again, had the disease been limited to a small number of individuals in Gibraltar? but there it was a frightful epidemic, and at two paces from thence not a single case of it.

This fact, absolutely inexplicable on the principles of local origin, considered independently of infection,† would become still more inexplicable, if I may use the expression, according to the doctrines of this latter system; and, in fact, how can the infectionists explain the matter? Why, in no other way than by saying, that causes of infection exist at Gibraltar, whilst no such causes exist in the neighbouring towns. Now, a state of things exactly the reverse of this obtains; that is to

* I shall say nothing of a town still nearer to Gibraltar, St. Roque, on account of its elevation above the level of the sea: an elevation inconsiderable, however, in comparison with that at which the yellow fever has been seen, not only in America, but also in Europe. Suffice it to say, that during the whole reign of the disease at Gibraltar, the population of St. Roque, as well as the Gibraltar families who had taken refuge there, before the establishment of the cordon, enjoyed the most perfect health.—AUTHOR.

† *Infection* is here restricted, as it is by almost all the French nosographers, to the production of disease, resulting to man in health, from breathing an atmosphere vitiated by certain emanations from the earth, stagnant water, drains, privies, and other sources of unwholesome effluvia.

By the term *contagion*, these writers mean to express the communication of the identical disease under which one or more individuals are actually labouring, or have lately laboured, to others placed in immediate or mediate contact with them, or with the things that surround, or have surrounded them, or to those obliged to breathe an atmosphere vitiated by emanations from these sick persons, or things, or from both. In short, infection is referred to inanimate matter, as its primary source; contagion to the living sick. It does not seem to be precisely determined, whether undue accumulation of persons in health, and meteoric causes, are included under the term infection or not. With our English writers, these two terms are generally used as if they were synonymous.—TRANSLATOR.

say, no causes of infection exist in Gibraltar, whereas such causes do exist in the neighbouring towns.

I shall not repeat here, what I have said already as to the extreme cleanliness of Gibraltar, in my "Notice Médicale" on that city: I shall merely express my conviction, that the world will not be likely to set down, as sufficient sources of infection, a few drains to be met with in the more sloping parts of the town, such as are found in our best policed cities; inconveniences at all times inseparable from a numerous population.

As to the towns near Gibraltar, it would not be difficult to discover in them causes calculated to produce a focus of infection. I shall mention only one town, Tariffa; but then it is a model in its way. The streets of Tariffa, generally narrow and unpaved, are strewed with filth of every kind; a canal, which with difficulty throws its contents into the sea, traverses the town from side to side: this canal, or, to use a more appropriate expression, this *cloaque*, the receptacle of all the sewers of the town, the banks of which are constantly covered with animals in a state of putrefaction, exhales, at all times, but particularly in the hot season, a smell that suffocates those who pass near to it, and incommodes the whole population. Thus might it be said, with truth, that Tariffa is the most infected town in the world: yet, (what is still totally inexplicable, agreeably to the doctrines of infection!) the yellow fever, which has often prevailed in Cadiz and Gibraltar, has never been seen at Tariffa, which stands midway between these two cities. The reason of this would, doubtless appear very simple to the partisans of exotic origin: Tariffa having no intercourse with America, whilst Cadiz and Gibraltar have. On the subject of Cadiz, I shall beg leave to add that, very like Gibraltar with regard to its advanced position in the sea, its elevation above the level of the water, &c., it is even better ventilated than the latter city, no breeze of wind being intercepted by its environs: yet this circumstance, no doubt very favorable to it upon the principles of infection, has, nevertheless, not saved it from being afflicted by the yellow fever, quite as often as Gibraltar.*

On the other hand, if the disease did not show itself in the neighbouring towns, when it prevailed in Gibraltar, is it not because it was introduced into the latter place, and not into the former. Besides we know, that not only did the disease

* Since the commencement of the nineteenth century, the yellow fever has appeared five times at Gibraltar, viz. in 1804, 1810, 1813, 1814, and 1828; and six times at Cadiz, in 1800, 1803, 1804, 1810, 1813, and 1819.—Author.

not shew itself in the neighbouring towns, at the times that it prevailed in Gibraltar, but that it has not appeared in these towns since. Now, as the towns alluded to took immediate measures against the introduction of the disease, as soon as it appeared, might not their having escaped it be owing to these measures? We know that the yellow fever has committed frequent ravages along the whole southern coast of the Peninsula; but what no one, I believe, has yet remarked, is that on the opposite coast, the northern coast of Africa, where the same soil, the same productions, the same atmospheric constitution, are found, the disease has never shewn itself.* This fact, it is needless to say, not only argues in favor of the exotic origin of the Gibraltar epidemic, but also in favor of the exotic origin of all the epidemics of the Peninsula. It is, indeed, evident, that it cannot be explained on any other grounds than that the Spanish coast has intercourse with America, and the African coast has none.

I shall close these reflections on the origin of the Gibraltar epidemic of 1828, by remarking,

1st. That the opinion as to its exotic origin was almost unanimous amongst the physicians of Gibraltar, English as well as Spanish; in which opinion the most enlightened and estimable persons of the garrison participated. Of these, I shall designate only the governor of the fortress, his Excellency General Sir George Don, and our own consul-general, M. Sylvestre de Sacy, son of the distinguished *savant* of that name.

2d. That the yellow fever prevailed from the beginning of the year, at different points of the West Indies, and of the continent of America; that many vessels, which in the course of the year passed the Straits, coming from these parts, had had sick and dead there; that the disease had been continued on board many of these vessels, so that some of them had persons still sick on board, even in the Straits. Such, amongst others, were two or three of the four ships which quitted the roads of Cadiz, on the 8th September, escorted by the king's brig, the Lynx.†

* Plague and typhus have often raged on this coast, but the yellow fever never. This I ascertained, during the voyage I made in July 1828. It is proper to mention, however, that, on the authority of some English physicians, M. Moreau de Jonnes notices the yellow fever as having occurred at Penon de Valez, in the year 1804.—Author.

† The ship Rosela, from the Havannah; the Louise, from Martinique; the Bernard and the Auguste, from Guadaloupe. The Rosela arrived on the 3d, the Bernard and the Auguste on the 2d, had had sick and dead where they came from, or on the passage; the Louise alone arrived on the 7th, had only sick. Dr. Ameler, who was sent to inspect these ships by the board of health,

3d. That a ship from the Havannah, when the vessel had both sick and dead, has been generally accused of the importation of the disease; and that, though it had been discovered afterwards (which I am ignorant of,) that the disease existed before the arrival of this ship,* it would not be difficult, seeing the very active commerce kept up by Gibraltar with the whole of America, to prove that other vessels had arrived from the parts of the world alluded to, anteriorly to the breaking out of the disease.†

4th, and lastly. That, if the yellow fever be contagious, and consequently capable of being imported, it is not at all necessary, in order that a ship should introduce this disease, that she should actually *have*, or even *have had*, sick on board her. It is enough that she had been in places where the disease existed, had existed, or might have existed, and consequently it would be wrong to deny, as some have already done, the importation of the disease attributed to a particular ship, because that ship had no sick at the time, or because she might not have had any sick previously, or even because the disease did not exist at the place of her departure.

recognized the yellow fever in many of the sick which they still had on board. Dr. Ameler, having seen all the epidemics by which Cadiz had been afflicted since 1800, it cannot be imagined that he could have been mistaken as to the nature of the disease. This is the same Dr. Ameler who already enjoyed a well-earned reputation, at the time the first commission was sent to the Peninsula by our government, and who has been mentioned with eulogy by Professor Berthe, in the work which he published, as one of the members of that commission.—Author.

* The ship accused of having imported the disease sailed from the Havannah on the 12th of May, and arrived in Gibraltar on the 28th of June. The nature of her declaration *(patentie)* having subjected her to a quarantine of forty days, she was not admitted to free pratique before the 6th of August. The medical men of Gibraltar, with whom I was in correspondence during the epidemic, agreed in stating that the disease did not show itself before the latter end of August. But supposing that it had appeared previously to the 6th of August, must it also be supposed that, from the 28th of June, the day on which the ship arrived, to the 6th of August, the day on which her quarantine ceased, the crew had no communication with the town? To those who know how quarantines in general are observed, but more particularly to those who know how they are observed in Gibraltar, the existence of which almost entirely depends on smuggling, the answer would not be doubtful. In reference to this point, I shall state a fact, of a Cadiz merchant, who, at one of his own *soirées*, distributed amongst his friends cigars brought to him from the Havannah, by a ship which, that very morning, had been placed under the most rigorous quarantine.—Author.

† I am aware that this may be considered an arbitrary mode of argument; but we are compelled to admit it, if indispensable to the interpretation of facts.—Author.

